# The Surgical Treatment of Neurotic Conditions*Read before the Southwest Texas District Medical Association at the San Antonio meeting March 13, 1917.

**Published:** 1917-06

**Authors:** Jno. W. Kenney

**Affiliations:** San Antonio, Texas


### TEXAS MEDICAL JOURNAL

DR. F. E. DANIEL,
Founder.

MRS. F. E. DANIEL,
Publisher and Managing
Editor.

PUBLISHED MONTHLY.
Subscription:
$1.00 a Year.

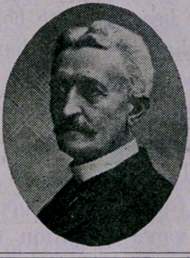

Established July,
1885.

VOL. XXXII No. 12
AUSTIN,
JUNE, 1917.

The publisher is not responsible for views of contributors.

*"All of good the past hath had,
Remains to make our own time glad."*—WHITTIER.

### Original Articles.

### The Surgical Treatment of Neurotic Conditions.*

BY JNO. W. KENNEY, M. D., SAN ANTONIO, TEXAS.

The aim of the author in the preparation of this short paper is to state facts as observed by him in treating neurotic conditions surgically. The etiology of many of these cases being often obscure, and the pathology being not always clear, has made it necessary to act in what would appear to be an empirical manner in many instances. An attempt has therefore been made to be practical, not necessarily scientific—to treat the subject from the standpoint of a general surgeon and not from that of the exclusive scientific specialist. Parenthetically, however, I wish to state that I believe, all such questions can be solved scientifically, provided we are possessed of sufficient common sense.

This paper is based upon a study of fifty cases, consisting of two thieves, two forgers, six congenital idiots, fifteen insane and twenty-five drunkards. This limited number has been selected because a number of years have passed since they were operated upon and their present condition can be considered final so far as the results of the operations are concerned. The operative procedures on the idiots, thieves and forgers were decompression operations. The results obtained do not justify going into details.

*Read before the Southwest Texas District Medical Association at the San Antonio meeting March 13, 1917.

They were failures so far as benefiting the patients were concerned. These interesting observations, however, were made:

In the case of the thieves and forgers, no changes were observed, except the possible variation in the method of plying their vocations. The thieves became more bold in their work, reasoning, I am confident, that the fact that I operated upon them, would assist them to escape punishment when caught. The forgers adopted the interesting method of forging my name to checks and getting some of my medical friends to cash them.

The idiots in several instances were apparently greatly benefited for a short time following the operation. At present they are practically in the same condition as before being operated upon. Those apparently slightly benefited would probably have developed to an equal extent had they never been treated surgically. The cases operated upon for different forms of insanity and habitual drunkenness present an entirely different picture.

The results obtained were most gratifying to myself and likewise to the patients. We find definite lesions in many of these usually considered hopeless cases on which to base our operative procedure. We, therefore, do not have to act empirically as in the class of patients previously mentioned in this paper.

I believe we are justified, however, in operating to break up the chain of morbid phenomena in many of these cases where a definite lesion capable of reflexly producing morbid conditions is not discernible. Of the three male and twelve female insane patients considered in the preparation of this paper, six are practically sane and the remainder with three exceptions are able to care for themselves.

The operations performed were two castrations, two trephinings, six panhysterectomies and five oophorectomies. One oophorectomy case was a failure, although there was a definite lesion; am confident the patient would have recovered had the uterus been removed at the same time. Of the panhysterectomies, one was a complete failure, notwithstanding the fact she became sane shortly after the operation and remained so for over a year. She is at present insane. The other failure recorded is that of a middle-aged man. He was castrated for a lesion of the testicle, with the hope that it would improve his mental condition.

If the result of these operations had been reversed and only three instead of twelve out of the fifteen been benefited, it is my opinion that the operative procedures would have been justifiable. The case of Mrs. M., a multipara of French ancestry, fairly illustrates the successfully treated cases. She had been ill a number of months, had been treated medicinally by medical men of unquestioned ability, and as a last resort was legally pronounced insane. She gave a history of having had a miscarriage just previous to the beginning of her illness and upon examination her genital organs were found enlarged and tender. She was noisy and violent to such an extent that the hospitals of the city would not receive her. A room in a private home was secured and an oophorectomy performed September, 1903. It was three days before the woman realized she had been operated upon. The recovery of her mental faculties was rapid and complete. She returned home within three weeks of the date of the operation and has conducted her household affairs and enjoyed good health both mentally and physically from that date to the present time.

The twenty-five cases referred to as having been operated upon for the cure of drunkenness or chronic alcoholism were so treated between the years 1908 and 1912. Of this number, ten have remained total abstainers, six have been intoxicated at irregular intervals, but not to such an extent as to incapacitate them from business, while the remainder have returned to their old condition of habitual drunkenness.

The first patient operated upon for this condition was a tramp about the streets of San Antonio for four years previous to this treatment. Two months after his operation he secured a position in a well know mercantile establishment and holds the same position to the present time. He was 35 years old; had been drinking to excess for ten years, the last four of which he was considered past redemption and absolutely valueless from a business standpoint.

Another interesting case is that of a bartender in one of the largest saloons in the city. He was about 30 years of age; had been drinking for a number of years. During the last year of this drinking period he had been subject to epileptic seizures at intervals of one or two weeks. After being operated upon, his epileptic seizures disappeared, and although he has continued to attend bar he has not been intoxicated.

The operation performed in these cases is a simple posterior gastrojejunostomy. My reason for resorting to this operation for the cure of temulentia is because of pathological condition produced in the stomach by alcohol is almost identical with the pathological conditions produced by other causes which are relieved by surgical treatment. That the neurotic conditions produced by alcohol are secondary to the condition found in the stomach "is very evident" to one who has watched the disappearance of these symptoms after the performance of a gastrojejunostomy. The technique of this operation becomes more and more simple as one becomes accustomed to its performance.

At present the writer never uses clamps and often does the work with a local anesthetic. After more than eight years' experience with this operation for the relief of chronic alcoholism, I am confident that 40 or 50 per cent of these cases would be permanently cured by its performance and many more improved. If 5 per cent should remain well, it would in the opinion of many be justifiable.

The percentage of recoveries after an operation for neurotic conditions can be increased very decidedly by restricting our work to selected cases. If one should restrict his work in the cases of insanity due to the diseases of the genital glands, to those in which there was obvious disease of these organs, his percentage of recoveries would increase. Should one refuse to operate upon all drunkards, except those with an occupation, good environments and gentlemanly instincts, his percentage of recoveries would be materially increased.

In both instances, however, we would be deprived of the satisfaction which comes to one who has helped the hopeless and made life worth living for some otherwise miserable human being. In these cases, as in all others, removal of the cause is the essential factor—the method of doing so is of secondary importance.

---

The members of the South Texas District Medical Association have chosen Beaumont as their next meeting place.

---

There will be no summer school for Baylor medical students this summer, as a request from the Chairman of the Council of National Medical Defense.

---

The State Board of Health has established a bureau of rural sanitation to direct, supervise and assist in conducting the health campaigns of the rural districts. The State Legislature recently appropriated $70,000 to carry on the work, and Dr. P. W. Covington of the University of Texas has been selected as the head of the new department.

